# Supplementary material for: MICAFlow: Fast and robust MRI preprocessing bridging research neuroimaging and clinical practice
Source: Imaging Neurosci (Camb). 2026 Sep 11;4:IMAG.a.1366. doi: 10.1162/IMAG.a.1366 (PMC13576946; doi:10.1162/IMAG.a.1366)
Supplement: Supplementary file 1 [file IMAG.a.1366_supp.pdf]

Supplementary Table 1

**CPU**	AMD Ryzen 9 7900X
**Single Core Clock Speed**	4.7 GHz (5.6 GHz Boost)
**Number of cores**	12 core (24 thread)
**RAM**	64 GB
**RAM Speed**	4800 MHz
**Hard Drive**	WD_BLACK SN750
**Hard Drive Read/Write**	Up to 3600 MB/s R, 2900 MB/s W

SUPPLEMENTARY TABLE 2

REGISTRATION	METRIC	COMP	X̄ REF	X̄ COMP	T_STAT	P_RAW	P_ADJ
T1w/ T1w	MI	ANTs	-0.52201	-0.52124	-1.06158	0.289	0.289
T1w/T1w	MI	EasyReg	-0.52201	-0.46908	-12.5824	1.779E-29	3.557E-29
T1w/T1w	MIND	ANTs	0.009117	0.00917	-3.10943	0.002	0.003
T1w/T1w	MIND	EasyReg	0.009117	0.01055	-14.1622	2.880E-35	1.728E-34
T1w/T1w	NGF	ANTs	-0.04654	-0.04637	-1.95072	0.052	0.062
T1w/T1w	NGF	EasyReg	-0.04654	-0.04045	-13.254	6.447E-32	1.934E-31
FLAIR/T1w	MI	ANTs	-0.44022	-0.4401	-0.73099	0.466	0.560
FLAIR/T1w	MI	EasyReg	-0.44022	-0.42769	-49.4597	4.694E-75	2.816E-74
FLAIR/T1w	MIND	ANTs	0.010494	0.01059	-22.3731	6.000E-42	1.200E-41
FLAIR/T1w	MIND	EasyReg	0.010494	0.01049	0.3602	0.719	0.719
FLAIR/T1w	NGF	ANTs	-0.03964	-0.03919	-25.6597	2.784E-47	8.351E-47
FLAIR/T1w	NGF	EasyReg	-0.03964	-0.03958	-2.34862	0.021	0.031
dMRI/T1w	MI	ANTs	-0.38967	-0.37782	-19.7499	6.195E-54	9.292E-54
dMRI/T1w	MI	FSL	-0.38967	-0.36093	-30.4175	2.896E-88	2.606E-87
dMRI/T1w	MI	EasyReg	-0.38967	-0.37622	-12.4201	3.354E-28	3.354E-28
dMRI/T1w	MIND	ANTs	0.01180	0.01229	-20.9608	4.137E-58	7.447E-58
dMRI/T1w	MIND	FSL	0.01180	0.01238	-24.9055	2.943E-71	8.829E-71
dMRI/T1w	MIND	EasyReg	0.01180	0.01213	-12.5759	9.789E-29	1.101E-28
dMRI/T1w	NGF	ANTs	-0.03596	-0.03395	-23.033	4.127E-65	9.285E-65
dMRI/T1w	NGF	FSL	-0.03596	-0.03304	-27.1452	2.286E-78	1.029E-77
dMRI/T1w	NGF	EasyReg	-0.03596	-0.0339	-14.4172	3.764E-35	4.840E-35

SUPPLEMENTARY TABLE 3

MODALITY	AUG	STRENGTH	METRIC	METHOD	X̄ LAMAREG	X̄ REF	X̄ DIFF	P_RAW	P_ADJ
T1W	NOISE	BASELINE	MI	ANTS (A)	-0.52201	-0.52124	-0.00077	0.289273	0.289273
T1W	NOISE	BASELINE	MI	EASYREG (E)	-0.52201	-0.46908	-0.05293	1.78E-29	3.56E-29
T1W	NOISE	2.50%	MI	ANTS (A)	-0.52279	-0.52188	-0.00091	2.24E-45	4.47E-45
T1W	NOISE	2.50%	MI	EASYREG (E)	-0.52457	-0.48142	-0.04315	1.05E-16	1.05E-16
T1W	NOISE	5%	MI	ANTS (A)	-0.52589	-0.52485	-0.00104	8.87E-09	8.87E-09
T1W	NOISE	5%	MI	EASYREG (E)	-0.52599	-0.48222	-0.04376	5.11E-23	1.02E-22
T1W	NOISE	10%	MI	ANTS (A)	-0.44364	-0.52024	0.0766	1.76E-16	3.51E-16
T1W	NOISE	10%	MI	EASYREG (E)	-0.44313	-0.47226	0.029128	2.62E-05	2.62E-05
T1W	MOTION	BASELINE	MI	ANTS (A)	-0.52201	-0.52124	-0.00077	0.289273	0.289273
T1W	MOTION	BASELINE	MI	EASYREG (E)	-0.52201	-0.46908	-0.05293	1.78E-29	3.56E-29
T1W	MOTION	5DEG	MI	ANTS (A)	-0.49836	-0.49936	0.000996	9.94E-06	9.94E-06
T1W	MOTION	5DEG	MI	EASYREG (E)	-0.49836	-0.49112	-0.00724	9.63E-44	1.93E-43
T1W	MOTION	10DEG	MI	ANTS (A)	-0.50324	-0.50417	0.00093	1.29E-07	1.29E-07
T1W	MOTION	10DEG	MI	EASYREG (E)	-0.50324	-0.49233	-0.0109	3.61E-10	7.21E-10
T1W	MOTION	20DEG	MI	ANTS (A)	-0.50325	-0.50396	0.000706	0.01905	0.01905
T1W	MOTION	20DEG	MI	EASYREG (E)	-0.50325	-0.47723	-0.02602	1.60E-18	3.19E-18
FLAIR	NOISE	BASELINE	MI	ANTS (A)	-0.44022	-0.4401	-0.00012	0.466396	0.466396
FLAIR	NOISE	BASELINE	MI	EASYREG (E)	-0.44022	-0.42769	-0.01253	4.69E-75	9.39E-75
FLAIR	NOISE	2.50%	MI	ANTS (A)	-0.43973	-0.43912	-0.0006	0.000314	0.000314
FLAIR	NOISE	2.50%	MI	EASYREG (E)	-0.43973	-0.42766	-0.01207	7.83E-74	1.57E-73
FLAIR	NOISE	5%	MI	ANTS (A)	-0.43931	-0.43855	-0.00076	4.34E-05	4.34E-05
FLAIR	NOISE	5%	MI	EASYREG (E)	-0.43931	-0.40711	-0.0322	1.93E-12	3.86E-12
FLAIR	NOISE	10%	MI	ANTS (A)	-0.42505	-0.43749	0.012447	0.054576	0.054576
FLAIR	NOISE	10%	MI	EASYREG (E)	-0.42505	-0.36862	-0.05642	7.29E-12	1.46E-11
FLAIR	MOTION	BASELINE	MI	ANTS (A)	-0.44022	-0.4401	-0.00012	0.466396	0.466396
FLAIR	MOTION	BASELINE	MI	EASYREG (E)	-0.44022	-0.42769	-0.01253	4.69E-75	9.39E-75
FLAIR	MOTION	5DEG	MI	ANTS (A)	-0.42845	-0.42839	-6.34E-05	0.784845	0.784845
FLAIR	MOTION	5DEG	MI	EASYREG (E)	-0.42845	-0.42027	-0.00819	7.99E-49	1.60E-48
FLAIR	MOTION	10DEG	MI	ANTS (A)	-0.42606	-0.42629	0.000233	0.674103	0.674103
FLAIR	MOTION	10DEG	MI	EASYREG (E)	-0.42606	-0.41848	-0.00757	1.76E-29	3.51E-29
FLAIR	MOTION	20DEG	MI	ANTS (A)	-0.42771	-0.42769	-2.43E-05	0.974687	0.974687
FLAIR	MOTION	20DEG	MI	EASYREG (E)	-0.42771	-0.41741	-0.0103	1.28E-27	2.57E-27
DMRI	NOISE	BASELINE	MI	ANTS (A)	-0.38967	-0.37782	-0.01184	6.19E-54	9.29E-54
DMRI	NOISE	BASELINE	MI	EASYREG (E)	-0.38967	-0.37622	-0.01345	3.35E-28	3.35E-28
DMRI	NOISE	BASELINE	MI	FSL (F)	-0.38967	-0.36093	-0.02873	2.90E-88	8.69E-88
DMRI	NOISE	2.50%	MI	ANTS (A)	-0.39045	-0.37698	-0.01346	6.21E-61	9.32E-61
DMRI	NOISE	2.50%	MI	EASYREG (E)	-0.39022	-0.38025	-0.00998	2.67E-30	2.67E-30
DMRI	NOISE	2.50%	MI	FSL (F)	-0.39045	-0.36104	-0.02941	1.45E-88	4.35E-88
DMRI	NOISE	5%	MI	ANTS (A)	-0.39075	-0.3789	-0.01185	2.80E-55	4.20E-55
DMRI	NOISE	5%	MI	EASYREG (E)	-0.39075	-0.38176	-0.00899	4.99E-52	4.99E-52
DMRI	NOISE	5%	MI	FSL (F)	-0.39075	-0.36088	-0.02987	2.02E-88	6.06E-88
DMRI	NOISE	10%	MI	ANTS (A)	-0.3901	-0.37946	-0.01065	2.72E-47	2.72E-47
DMRI	NOISE	10%	MI	EASYREG (E)	-0.3901	-0.38196	-0.00814	2.60E-48	3.90E-48
DMRI	NOISE	10%	MI	FSL (F)	-0.3901	-0.3609	-0.02921	3.70E-84	1.11E-83
DMRI	MOTION	BASELINE	MI	ANTS (A)	-0.38967	-0.37782	-0.01184	6.19E-54	9.29E-54

DMRI	MOTION	BASELINE	MI	EASYREG (E)	-0.38967	-0.37622	-0.01345	3.35E-28	3.35E-28
DMRI	MOTION	BASELINE	MI	FSL (F)	-0.38967	-0.36093	-0.02873	2.90E-88	8.69E-88
DMRI	MOTION	5DEG	MI	ANTS (A)	-0.37932	-0.36803	-0.01129	6.72E-37	1.01E-36
DMRI	MOTION	5DEG	MI	EASYREG (E)	-0.37897	-0.37291	-0.00606	1.15E-06	1.15E-06
DMRI	MOTION	5DEG	MI	FSL (F)	-0.37932	-0.35892	-0.0204	1.81E-52	5.42E-52
DMRI	MOTION	10DEG	MI	ANTS (A)	-0.3799	-0.36788	-0.01202	1.64E-46	4.93E-46
DMRI	MOTION	10DEG	MI	EASYREG (E)	-0.3799	-0.37539	-0.00451	0.006661	0.006661
DMRI	MOTION	10DEG	MI	FSL (F)	-0.3799	-0.36004	-0.01986	9.76E-44	1.46E-43
DMRI	MOTION	20DEG	MI	ANTS (A)	-0.37872	-0.36563	-0.01309	2.21E-33	6.62E-33
DMRI	MOTION	20DEG	MI	EASYREG (E)	-0.37804	-0.3713	-0.00674	0.000548	0.000548
DMRI	MOTION	20DEG	MI	FSL (F)	-0.37872	-0.36096	-0.01776	2.02E-31	3.03E-31
T1W	NOISE	BASELINE	MIND	ANTS (A)	0.009117	0.009169	-5.22E-05	0.002053	0.002053
T1W	NOISE	BASELINE	MIND	EASYREG (E)	0.009117	0.010552	-0.00143	2.88E-35	5.76E-35
T1W	NOISE	2.50%	MIND	ANTS (A)	0.009119	0.009173	-5.44E-05	3.43E-64	6.87E-64
T1W	NOISE	2.50%	MIND	EASYREG (E)	0.009231	0.010451	-0.00122	3.08E-21	3.08E-21
T1W	NOISE	5%	MIND	ANTS (A)	0.00938	0.009436	-5.59E-05	3.07E-30	6.14E-30
T1W	NOISE	5%	MIND	EASYREG (E)	0.009381	0.010865	-0.00148	3.25E-27	3.25E-27
T1W	NOISE	10%	MIND	ANTS (A)	0.010479	0.009258	0.001221	6.03E-17	1.21E-16
T1W	NOISE	10%	MIND	EASYREG (E)	0.010479	0.011065	-0.00059	3.94E-10	3.94E-10
T1W	MOTION	BASELINE	MIND	ANTS (A)	0.009117	0.009169	-5.22E-05	0.002053	0.002053
T1W	MOTION	BASELINE	MIND	EASYREG (E)	0.009117	0.010552	-0.00143	2.88E-35	5.76E-35
T1W	MOTION	5DEG	MIND	ANTS (A)	0.009817	0.009784	3.30E-05	3.96E-05	3.96E-05
T1W	MOTION	5DEG	MIND	EASYREG (E)	0.009817	0.010023	-0.00021	8.11E-27	1.62E-26
T1W	MOTION	10DEG	MIND	ANTS (A)	0.009917	0.00989	2.68E-05	2.25E-06	2.25E-06
T1W	MOTION	10DEG	MIND	EASYREG (E)	0.009917	0.010145	-0.00023	8.31E-10	1.66E-09
T1W	MOTION	20DEG	MIND	ANTS (A)	0.009908	0.009887	2.10E-05	0.008416	0.008416
T1W	MOTION	20DEG	MIND	EASYREG (E)	0.009908	0.01047	-0.00056	2.28E-16	4.56E-16
FLAIR	NOISE	BASELINE	MIND	ANTS (A)	0.010494	0.010592	-9.78E-05	6.00E-42	1.20E-41
FLAIR	NOISE	BASELINE	MIND	EASYREG (E)	0.010494	0.010491	3.44E-06	0.7194	0.7194
FLAIR	NOISE	2.50%	MIND	ANTS (A)	0.010509	0.010596	-8.77E-05	1.15E-45	2.29E-45
FLAIR	NOISE	2.50%	MIND	EASYREG (E)	0.010509	0.010487	2.12E-05	0.028987	0.028987
FLAIR	NOISE	5%	MIND	ANTS (A)	0.01052	0.010618	-9.83E-05	1.95E-45	3.91E-45
FLAIR	NOISE	5%	MIND	EASYREG (E)	0.01052	0.011095	-0.00057	1.68E-13	1.68E-13
FLAIR	NOISE	10%	MIND	ANTS (A)	0.010622	0.010655	-3.32E-05	0.243282	0.243282
FLAIR	NOISE	10%	MIND	EASYREG (E)	0.010622	0.012317	-0.00169	2.21E-30	4.42E-30
FLAIR	MOTION	BASELINE	MIND	ANTS (A)	0.010494	0.010592	-9.78E-05	6.00E-42	1.20E-41
FLAIR	MOTION	BASELINE	MIND	EASYREG (E)	0.010494	0.010491	3.44E-06	0.7194	0.7194
FLAIR	MOTION	5DEG	MIND	ANTS (A)	0.010903	0.010976	-7.36E-05	2.90E-11	5.81E-11
FLAIR	MOTION	5DEG	MIND	EASYREG (E)	0.010903	0.010849	5.33E-05	0.002744	0.002744
FLAIR	MOTION	10DEG	MIND	ANTS (A)	0.010971	0.011012	-4.12E-05	0.008163	0.008163
FLAIR	MOTION	10DEG	MIND	EASYREG (E)	0.010971	0.010909	6.22E-05	0.007616	0.008163
FLAIR	MOTION	20DEG	MIND	ANTS (A)	0.010918	0.010992	-7.44E-05	0.020627	0.041253
FLAIR	MOTION	20DEG	MIND	EASYREG (E)	0.010918	0.010944	-2.61E-05	0.475827	0.475827
DMRI	NOISE	BASELINE	MIND	ANTS (A)	0.011803	0.012293	-0.00049	4.14E-58	6.21E-58
DMRI	NOISE	BASELINE	MIND	EASYREG (E)	0.011803	0.012137	-0.00033	9.79E-29	9.79E-29
DMRI	NOISE	BASELINE	MIND	FSL (F)	0.011803	0.012375	-0.00057	2.94E-71	8.83E-71
DMRI	NOISE	2.50%	MIND	ANTS (A)	0.011807	0.012287	-0.00048	1.34E-54	2.01E-54
DMRI	NOISE	2.50%	MIND	EASYREG (E)	0.011797	0.011984	-0.00019	1.64E-19	1.64E-19

DMRI	NOISE	2.50%	MIND	FSL (F)	0.011807	0.012375	-0.00057	4.39E-70	1.32E-69
DMRI	NOISE	5%	MIND	ANTS (A)	0.011822	0.0123	-0.00048	1.50E-55	2.26E-55
DMRI	NOISE	5%	MIND	EASYREG (E)	0.011822	0.011933	-0.00011	1.98E-31	1.98E-31
DMRI	NOISE	5%	MIND	FSL (F)	0.011822	0.012375	-0.00055	3.01E-67	9.04E-67
DMRI	NOISE	10%	MIND	ANTS (A)	0.011887	0.012354	-0.00047	1.00E-54	1.50E-54
DMRI	NOISE	10%	MIND	EASYREG (E)	0.011887	0.011959	-7.14E-05	3.54E-12	3.54E-12
DMRI	NOISE	10%	MIND	FSL (F)	0.011887	0.012377	-0.00049	5.55E-57	1.67E-56
DMRI	MOTION	BASELINE	MIND	ANTS (A)	0.011803	0.012293	-0.00049	4.14E-58	6.21E-58
DMRI	MOTION	BASELINE	MIND	EASYREG (E)	0.011803	0.012137	-0.00033	9.79E-29	9.79E-29
DMRI	MOTION	BASELINE	MIND	FSL (F)	0.011803	0.012375	-0.00057	2.94E-71	8.83E-71
DMRI	MOTION	5DEG	MIND	ANTS (A)	0.012189	0.012673	-0.00048	4.30E-64	1.29E-63
DMRI	MOTION	5DEG	MIND	EASYREG (E)	0.012191	0.01236	-0.00017	1.92E-11	1.92E-11
DMRI	MOTION	5DEG	MIND	FSL (F)	0.012189	0.012485	-0.0003	4.96E-28	7.45E-28
DMRI	MOTION	10DEG	MIND	ANTS (A)	0.012191	0.012706	-0.00052	1.11E-63	3.33E-63
DMRI	MOTION	10DEG	MIND	EASYREG (E)	0.012191	0.012396	-0.0002	2.34E-14	3.50E-14
DMRI	MOTION	10DEG	MIND	FSL (F)	0.012191	0.012415	-0.00022	5.11E-14	5.11E-14
DMRI	MOTION	20DEG	MIND	ANTS (A)	0.012204	0.01271	-0.00051	2.72E-59	8.17E-59
DMRI	MOTION	20DEG	MIND	EASYREG (E)	0.012173	0.012515	-0.00034	1.42E-28	2.13E-28
DMRI	MOTION	20DEG	MIND	FSL (F)	0.012204	0.012353	-0.00015	1.67E-06	1.67E-06
T1W	NOISE	BASELINE	NGF	ANTS (A)	-0.04654	-0.04637	-0.00017	0.052015	0.052015
T1W	NOISE	BASELINE	NGF	EASYREG (E)	-0.04654	-0.04045	-0.00609	6.45E-32	1.29E-31
T1W	NOISE	2.50%	NGF	ANTS (A)	-0.04659	-0.04642	-0.00017	2.45E-60	4.89E-60
T1W	NOISE	2.50%	NGF	EASYREG (E)	-0.04648	-0.0411	-0.00538	2.18E-18	2.18E-18
T1W	NOISE	5%	NGF	ANTS (A)	-0.04624	-0.04605	-0.00019	4.93E-17	4.93E-17
T1W	NOISE	5%	NGF	EASYREG (E)	-0.04625	-0.04067	-0.00559	1.68E-26	3.37E-26
T1W	NOISE	10%	NGF	ANTS (A)	-0.03818	-0.04616	0.007971	6.86E-17	1.37E-16
T1W	NOISE	10%	NGF	EASYREG (E)	-0.03819	-0.03936	0.001174	0.057772	0.057772
T1W	MOTION	BASELINE	NGF	ANTS (A)	-0.04654	-0.04637	-0.00017	0.052015	0.052015
T1W	MOTION	BASELINE	NGF	EASYREG (E)	-0.04654	-0.04045	-0.00609	6.45E-32	1.29E-31
T1W	MOTION	5DEG	NGF	ANTS (A)	-0.04388	-0.04397	8.45E-05	0.02589	0.02589
T1W	MOTION	5DEG	NGF	EASYREG (E)	-0.04388	-0.04305	-0.00083	6.00E-36	1.20E-35
T1W	MOTION	10DEG	NGF	ANTS (A)	-0.0441	-0.04416	5.60E-05	0.02572	0.02572
T1W	MOTION	10DEG	NGF	EASYREG (E)	-0.0441	-0.04296	-0.00114	4.80E-11	9.60E-11
T1W	MOTION	20DEG	NGF	ANTS (A)	-0.04415	-0.04417	2.69E-05	0.503604	0.503604
T1W	MOTION	20DEG	NGF	EASYREG (E)	-0.04415	-0.04133	-0.00281	3.75E-19	7.51E-19
FLAIR	NOISE	BASELINE	NGF	ANTS (A)	-0.03964	-0.03919	-0.00045	2.78E-47	5.57E-47
FLAIR	NOISE	BASELINE	NGF	EASYREG (E)	-0.03964	-0.03958	-6.49E-05	0.020698	0.020698
FLAIR	NOISE	2.50%	NGF	ANTS (A)	-0.03958	-0.03917	-0.00041	2.07E-51	4.15E-51
FLAIR	NOISE	2.50%	NGF	EASYREG (E)	-0.03958	-0.03958	-2.81E-06	0.922233	0.922233
FLAIR	NOISE	5%	NGF	ANTS (A)	-0.03953	-0.03908	-0.00045	1.92E-50	3.85E-50
FLAIR	NOISE	5%	NGF	EASYREG (E)	-0.03953	-0.03681	-0.00272	1.66E-11	1.66E-11
FLAIR	NOISE	10%	NGF	ANTS (A)	-0.0385	-0.03893	0.000424	0.342254	0.342254
FLAIR	NOISE	10%	NGF	EASYREG (E)	-0.0385	-0.0309	-0.0076	1.06E-21	2.12E-21
FLAIR	MOTION	BASELINE	NGF	ANTS (A)	-0.03964	-0.03919	-0.00045	2.78E-47	5.57E-47
FLAIR	MOTION	BASELINE	NGF	EASYREG (E)	-0.03964	-0.03958	-6.49E-05	0.020698	0.020698
FLAIR	MOTION	5DEG	NGF	ANTS (A)	-0.03814	-0.03774	-0.00041	6.97E-14	1.39E-13
FLAIR	MOTION	5DEG	NGF	EASYREG (E)	-0.03814	-0.03823	8.44E-05	0.164747	0.164747
FLAIR	MOTION	10DEG	NGF	ANTS (A)	-0.03781	-0.03758	-0.00023	0.005352	0.010703

FLAIR	MOTION	10DEG	NGF	EASYREG (E)	-0.03781	-0.03791	9.92E-05	0.284174	0.284174
FLAIR	MOTION	20DEG	NGF	ANTS (A)	-0.03804	-0.03767	-0.00037	0.021805	0.032158
FLAIR	MOTION	20DEG	NGF	EASYREG (E)	-0.03804	-0.03768	-0.00036	0.032158	0.032158
DMRI	NOISE	BASELINE	NGF	ANTS (A)	-0.03596	-0.03395	-0.00201	4.13E-65	6.19E-65
DMRI	NOISE	BASELINE	NGF	EASYREG (E)	-0.03596	-0.0339	-0.00206	3.76E-35	3.76E-35
DMRI	NOISE	BASELINE	NGF	FSL (F)	-0.03596	-0.03304	-0.00292	2.29E-78	6.86E-78
DMRI	NOISE	2.50%	NGF	ANTS (A)	-0.03593	-0.03386	-0.00207	1.28E-62	1.92E-62
DMRI	NOISE	2.50%	NGF	EASYREG (E)	-0.03596	-0.03471	-0.00125	2.02E-29	2.02E-29
DMRI	NOISE	2.50%	NGF	FSL (F)	-0.03593	-0.03304	-0.00288	2.86E-76	8.57E-76
DMRI	NOISE	5%	NGF	ANTS (A)	-0.03585	-0.03382	-0.00203	4.78E-62	4.78E-62
DMRI	NOISE	5%	NGF	EASYREG (E)	-0.03585	-0.03501	-0.00084	3.76E-67	5.64E-67
DMRI	NOISE	5%	NGF	FSL (F)	-0.03585	-0.03305	-0.0028	1.44E-72	4.33E-72
DMRI	NOISE	10%	NGF	ANTS (A)	-0.03555	-0.03357	-0.00198	8.22E-60	1.23E-59
DMRI	NOISE	10%	NGF	EASYREG (E)	-0.03555	-0.03488	-0.00067	7.52E-38	7.52E-38
DMRI	NOISE	10%	NGF	FSL (F)	-0.03555	-0.03304	-0.0025	7.88E-62	2.36E-61
DMRI	MOTION	BASELINE	NGF	ANTS (A)	-0.03596	-0.03395	-0.00201	4.13E-65	6.19E-65
DMRI	MOTION	BASELINE	NGF	EASYREG (E)	-0.03596	-0.0339	-0.00206	3.76E-35	3.76E-35
DMRI	MOTION	BASELINE	NGF	FSL (F)	-0.03596	-0.03304	-0.00292	2.29E-78	6.86E-78
DMRI	MOTION	5DEG	NGF	ANTS (A)	-0.03396	-0.03204	-0.00191	1.58E-60	4.75E-60
DMRI	MOTION	5DEG	NGF	EASYREG (E)	-0.03395	-0.03276	-0.00119	1.09E-17	1.09E-17
DMRI	MOTION	5DEG	NGF	FSL (F)	-0.03396	-0.03251	-0.00145	2.12E-29	3.17E-29
DMRI	MOTION	10DEG	NGF	ANTS (A)	-0.03385	-0.03181	-0.00203	5.46E-53	1.64E-52
DMRI	MOTION	10DEG	NGF	EASYREG (E)	-0.03385	-0.03246	-0.00138	5.71E-21	8.56E-21
DMRI	MOTION	10DEG	NGF	FSL (F)	-0.03385	-0.03283	-0.00102	1.01E-11	1.01E-11
DMRI	MOTION	20DEG	NGF	ANTS (A)	-0.03357	-0.03151	-0.00205	3.45E-43	1.03E-42
DMRI	MOTION	20DEG	NGF	EASYREG (E)	-0.03358	-0.03111	-0.00247	3.49E-41	5.23E-41
DMRI	MOTION	20DEG	NGF	FSL (F)	-0.03357	-0.03297	-0.0006	0.000274	0.000274

SUPPLEMENTARY TABLE 4

DATASET	CATEGORY	MEAN	STD	MEAN_INTRA	MEAN_INTER
MICS	LINEAR	51.708	2.485	0.933	0.416
MICS	NONLINEAR	36.955	1.825	0.937	0.567
EPIC	LINEAR	40.828	2.463	0.890	0.482
EPIC	NONLINEAR	27.975	1.661	0.902	0.623
PNI	LINEAR	39.020	4.449	0.777	0.387
PNI	NONLINEAR	31.460	4.297	0.799	0.484
AMTRT	LINEAR	39.601	3.621	0.912	0.516
AMTRT	NONLINEAR	24.841	4.009	0.896	0.648

Supplementary Table 5

Dataset	Category	Metric	Mean	Std
MICs	T1_Linear	MI	-5.65E-01	4.10E-03
MICs	T1_SyN	MI	-6.00E-01	4.34E-03
MICs	FLAIR	MI	-4.29E-01	2.75E-02
MICs	dMRI	MI	-4.01E-01	2.49E-02
MICs	T1_Linear	MIND	2.04E-02	1.96E-04
MICs	T1_SyN	MIND	1.92E-02	2.10E-04
MICs	FLAIR	MIND	1.10E-02	1.11E-03
MICs	dMRI	MIND	1.29E-02	1.14E-03
MICs	T1_Linear	NGF	-5.31E-02	8.24E-04
MICs	T1_SyN	NGF	-5.87E-02	8.77E-04
MICs	FLAIR	NGF	-3.89E-02	3.12E-03
MICs	dMRI	NGF	-3.24E-02	2.86E-03
EpiC	T1_Linear	MI	-5.59E-01	1.10E-02
EpiC	T1_SyN	MI	-6.05E-01	6.05E-03
EpiC	FLAIR	MI	-4.11E-01	3.86E-02
EpiC	dMRI	MI	-3.40E-01	3.02E-02
EpiC	T1_Linear	MIND	2.02E-02	3.86E-04
EpiC	T1_SyN	MIND	1.86E-02	4.21E-04
EpiC	FLAIR	MIND	9.54E-03	1.38E-03
EpiC	dMRI	MIND	1.09E-02	1.50E-03
EpiC	T1_Linear	NGF	-5.46E-02	1.15E-03
EpiC	T1_SyN	NGF	-6.12E-02	1.46E-03
EpiC	FLAIR	NGF	-3.95E-02	5.41E-03
EpiC	dMRI	NGF	-2.78E-02	3.84E-03
PNI	T1_Linear	MI	-5.64E-01	3.00E-03
PNI	T1_SyN	MI	-5.93E-01	4.48E-03
PNI	dMRI	MI	-2.84E-01	3.40E-02

PNI	T1_Linear	MIND	2.05E-02	2.07E-04
PNI	T1_SyN	MIND	1.95E-02	2.22E-04
PNI	dMRI	MIND	1.47E-02	1.15E-03
PNI	T1_Linear	NGF	-5.19E-02	5.73E-04
PNI	T1_SyN	NGF	-5.65E-02	8.02E-04
PNI	dMRI	NGF	-2.98E-02	2.52E-03
AmTrT	T1_Linear	MI	-5.60E-01	1.02E-02
AmTrT	T1_SyN	MI	-6.00E-01	8.19E-03
AmTrT	dMRI	MI	-3.27E-01	2.71E-02
AmTrT	T1_Linear	MIND	2.01E-02	2.20E-04
AmTrT	T1_SyN	MIND	1.88E-02	2.36E-04
AmTrT	dMRI	MIND	1.09E-02	9.11E-04
AmTrT	T1_Linear	NGF	-5.48E-02	9.28E-04
AmTrT	T1_SyN	NGF	-6.14E-02	1.11E-03
AmTrT	dMRI	NGF	-3.05E-02	3.05E-03

SUPPLEMENTARY TABLE 6

DATASET	CATEGORY	MEAN	STD
MICS	FA	0.931173	1.61E-02
MICS	ADC	0.916933	2.41E-02
EPIC	FA	8.67E-01	5.40E-02
EPIC	ADC	0.813109	6.47E-02
PNI	FA	0.643343	8.25E-02
PNI	ADC	0.776989	8.27E-02
AMTRT	FA	0.940533	1.79E-02
AMTRT	ADC	0.849046	8.10E-02

SUPPLEMENTARY TABLE 7

DATASET	SOURCE	GROUP	MEAN	STD
EPIC	LINEAR	CONTROL	0.890	0.033
EPIC	NONLINEAR	CONTROL	0.902	0.025
EPIC	LINEAR	PATIENT	0.862	0.038
EPIC	NONLINEAR	PATIENT	0.863	0.041
MICS	LINEAR	CONTROL	0.933	0.027
MICS	NONLINEAR	CONTROL	0.937	0.014
MICS	LINEAR	PATIENT	0.906	0.033
MICS	NONLINEAR	PATIENT	0.925	0.022

SUPPLEMENTARY TABLE 8

DATASET	SOURCE	GROUP	IDENTIFIABILITY
EPIC	LINEAR	PATIENT	41.243
EPIC	NONLINEAR	PATIENT	27.232
EPIC	LINEAR	CONTROL	40.828
EPIC	NONLINEAR	CONTROL	27.975
MICS	LINEAR	CONTROL	51.708
MICS	NONLINEAR	CONTROL	36.955
MICS	LINEAR	PATIENT	49.543
MICS	NONLINEAR	PATIENT	35.781
